# Parsing the Neural Mechanisms of Short-Term and Long-Term Associations in the Flanker Tasks: An ERP Analysis

**DOI:** 10.3389/fnbeh.2021.626907

**Published:** 2021-08-05

**Authors:** Wenwen Cheng, Qiao Huang, Ying Chen, Weipeng Dai, Liyan Cui, Sharui Shan, Zhuoming Chen, Shu Zhou

**Affiliations:** ^1^Department of Neurology, The First Affiliated Hospital of Jinan University, Guangzhou, China; ^2^Department of Neurology, Nanfang Hospital, Southern Medical University, Guangzhou, China; ^3^Department of Rehabilitation, Guangzhou Red-Cross Hospital of Jinan University, Guangzhou, China; ^4^Department of Ideological and Political Theory Teaching, Maoming Polytechnic, Maoming, China; ^5^Department of Neurology, Jiangmen Central Hospital, Jiangmen, China; ^6^Department of Rehabilitation, The First Affiliated Hospital of Jinan University, Guangzhou, China; ^7^Department of Rehabilitation, The First Affiliated Hospital of Guangdong Pharmaceutical University, Guangzhou, China

**Keywords:** flanker task, stimulus-stimulus conflict, stimulus-response conflict, events related potentials (ERP), cognition conflict

## Abstract

The neural mechanisms of cognitive conflicts within various flanker tasks are still unclear, which may be mixed with different effects of short-term associations and long-term associations. We applied a perceptual (color) flanker task and a symbolic (arrow) flanker task to 25 healthy young adults, while the event-related potentials (ERP) and behavioral performance were recorded. The former contains stimulus-stimulus conflict (SSC) of short-term memory (STM) associations, and the latter contains stimulus-response conflict (SRC) of long-term memory (LTM) associations. Both flanker tasks included congruent and incongruent conditions. The reaction time demonstrated the stimulus-response conflict effect in the arrow flanker task without the stimulus-stimulus conflict effect in the color flanker task. The ERP results showed SSC enhanced the frontocentral N2b without behavioral effects. SRC increased the frontocentral P2 but decreased the centroparietal P3b with prolonged reaction time. In the comparison between both tasks, the color flanker task elicited both the centroparietal N2b/N300 and the frontocentral N400, and the arrow flanker task increased the occipital N1. Our findings provide new evidence that different neural mechanisms underlie conflict effects based on different types of memory associations.

## Introduction

Some behavioral studies and dimensional overlap (DO) theory have shown that the conflict effect in the stimulus-response compatibility paradigms is derived either from the conflict between relevant and irrelevant stimulus dimensions (stimulus-stimulus conflict, SSC), or from the conflict between irrelevant stimulus dimensions and relevant response dimensions (stimulus-response conflict, SRC) (De Jong et al., 1994; Kornblum, 1994; Kornblum et al., 1999; Treccani et al., 2009). Based on the DO taxonomy, the conflict effects of the flanker task belong to the SSC type. However, depending on the type of stimulus and experimental design, the flanker task may also contain two types of conflicts (De Houwer, 2003). When perceptual materials such as letters and colors are used as stimulus materials for the flanker task, conflicts will occur between relevant and irrelevant stimulus dimensions and SSC generated; when symbolic materials such as arrows are used as stimulus materials for the flanker task, then the conflict will occur between irrelevant stimulus dimensions and relevant response dimensions and SRC caused.

Eriksen flanker tasks using color and an arrow contain SSC and SRC, respectively. Previous research has revealed the involvement of at least three event-related potentials (ERP) components for SSC and SRC (Larson et al., 2014). However, which ERP components induced by SSC and SRC in the flanker paradigm have not yet achieved consistent results. First, many studies reported SSC enhanced a frontal N2b that emerges approximately between 250 and 350 ms after the stimulus onset (Kopp et al., 1996; Clayson and Larson, 2011), which comes from the anterior cingulate cortex (ACC) and reflects stimulus competition and/or response selection (Van Veen and Carter, 2002; Forster et al., 2011; Kim et al., 2011; Larson et al., 2014). Second, a study using arrow stimuli suggested that SRC enhances a frontal P2 (Kałamała et al., 2018). The frontal P2 is typically associated with selective attentional processes engaged in stimulus evaluation (Luck et al., 1994; Gajewski et al., 2008). We also observed similar frontal P2 for SRC in addition to the frontal N2b enhancement for SSC using a perceptual flanker 2:1 mapping task (Zhou et al., 2019), in which two colors were associated with each response hand, respectively. Finally, some studies discovered SRC decreased the parietal P3b (Kałamała et al., 2018; Brunetti et al., 2019; Zhou et al., 2019). Consequently, which ERP components induced by SSC and SRC in the flanker paradigm have not yet achieved consistent results.

Even the same type of conflict caused by the flanker task may involve different neural processes. It was pointed out in a recently published paper that the flanker task employing oriented hands or arrows can cause two types of SRC: one is activation based on short-term memory (STM) and required active processing, and the other is activation based on long-term memory (LTM) and is an automatically activated process (Brunetti et al., 2019). In an ERP and fMRI study using the color flanker and Simon paradigm showed that the color flanker conflict trials induced a larger fronto-central N2b, with a time window of 260–360 ms after the stimulus onset (Frühholz et al., 2011). Source localization analysis of this N2b component found that it originated from the superior frontal gyrus and corresponds to the activation of anterior cingulate cortex (ACC), which indicated the process of conflict resolution. In this study, the conflict effect included the SSC and the SRC based on STM association; the observed enhanced N2b in frontocentral regions indicated the conflict resolution process. Similar results were observed in a recent published ERP study conducted by Zhou et al. (2019). Zhou et al. applied a perceptual flanker 2–1 mapping task to reveal the neural mechanism of stimulus and response conflict of STM, and demonstrated that the SRC based on STM also induced the fronto-central N2b effect, which suggested the process of conflict resolution. Nevertheless, in a large sample of participants, the study employed the arrow flanker task as a basic paradigm; there was no difference in the amplitude of N2b induced by consistent and inconsistent trials (Kałamała et al., 2018), which indicated that the SRC based on LTM did not elicit the signature N2b component of conflict. These results implied that the resolution of conflicts based on STM or LTM may involve different neural processing mechanisms. However, the evidence from the flanker task or variant in different studies may be task-specific due to different experimental designs, stimulus materials, and experimental purposes, and may have an impact on the existing results.

On the other hand, the stimulus of the perceptual flanker task (color/letter) and the symbolic flanker task (arrow) convey different information (Ristic and Kingstone, 2006; Olk et al., 2008). For instance, arrows in the symbolic flanker task as overlearned symbols of direction, the direction information conveyed by them, and the mapping to certain responses (for “ < ” press the left button; for “>” press the right button) are decoded very quickly and accurately, and possibly even involuntarily. Relatively, the stimulus of color in the perceptual flanker task contains color features without direction information; thus, the encoding process of stimulus-response mapping based on STM was slower and with more effort, and may involve the process of color information. Previous studies on conflict control that applied the perceptual flanker task have reported the relevant waveform, such as N2pc, N2b, and indicated that it is related to attention selection and conflict resolution (Kim et al., 2011; Cespón et al., 2013a,b; Larson et al., 2014). However, previous studies on color processing have shown that the perceptual flanker task when color as stimulus material might also involve the classification of color strategies. An ERP study applied four types of stimuli based on hue differences to investigate the nature of the linguistic effect on color perception and observed N2b occurring at 200–350 ms after the stimulus onset, which indicated the occurrence of color category effect (Lu et al., 2014). A left-lateralized N2pc was also found by using the color category task (Liu et al., 2009). The above results strongly implied that the category effect was also involved in the perceptual flanker task when color was stimulus material. Therefore, previous studies on the neural mechanism of conflict effects based on different types of memory associations (STM/LTM) in flanker tasks may ignore the influence of the color category effect.

Here, we attempt to separate the mixing factors by directly comparing the perceptual and the symbolic flanker task to explore the neural mechanism of the SSC effect based on STM and the SRC effect based on LTM. In the present study, we applied a color flanker task and an arrow flanker task; both of the two flanker tasks included congruent and incongruent conditions. Hence, there are four types of trials: arrow-congruent, arrow-incongruent, color-congruent, and color-incongruent. The color stimuli involve a color category, and color-based flanker stimuli contain SSC effects; the arrow as an overlearned symbol conveys direction information, and flanker stimuli based on arrow symbols contain SRC effects.

## Materials and Methods

### Participants

Twenty-five healthy undergraduate and graduate students (12 men; age range: 22–28 years; mean age: 25.16 years, SD: 1.55 years) served as paid participants. All were right-handed and had normal or corrected-to-normal vision without color blindness. None had a history of neurological or psychiatric disorders. The participants were fully informed about the schedule and goals of the study and gave written informed consent in accordance with procedures approved by the Medical Ethics Committee of Nanfang Hospital, Southern Medical University.

### Stimuli and Procedure

**Figure 1:**
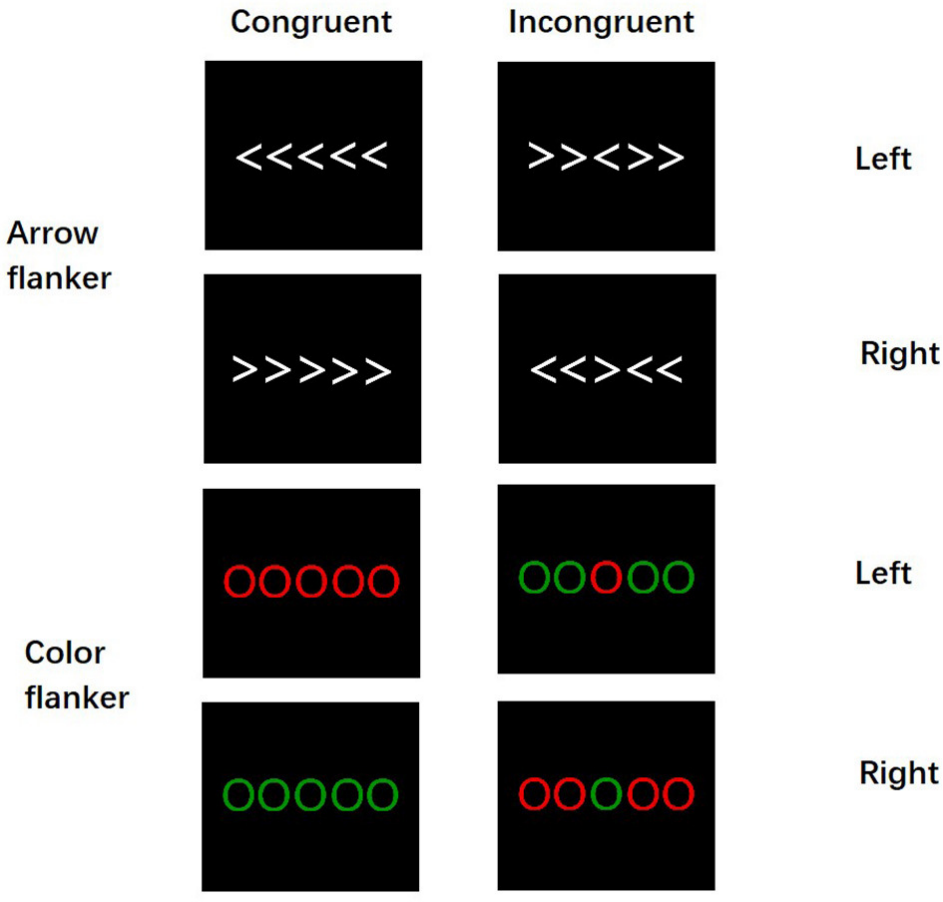
The arrow flanker task and the color flanker task.

The participants completed the flanker task in a sound-attenuated, dimly lit chamber. The stimuli were presented on the center of a 17-in computer monitor connected to a ThinkPad notebook. The distance between the screen and the participants was 120 cm. The target stimulus consisted of five parallel white arrows or five parallel-colored circles (red or green) (Figure 1). All target stimuli were presented in pseudorandom equal probability without fixations. White arrows and colored circles stimuli, each measuring 10 × 3 cm (5.3° horizontally and 0.65° vertically) in width and height, were presented against a dark gray background. The participants were asked to respond rapidly to the direction of the center arrow (if the arrow points left, press the left button; otherwise, press the right button) and the color of the center-colored circle (when the color is red, press the left button; when the color is green, press the right button) but without sacrificing accuracy. In congruent trials, the Flanker stimuli (the direction of the arrow or the colored circle on the side) were of the same color or the direction of the arrow as the center stimuli, whereas, in incongruent trials, the color or the direction of the central arrow did not match the flanker stimuli; thereby, there were four types of stimulus: (1) the congruent trials in the arrow flanker task (Arr-Con), (2) the incongruent trials in the arrow flanker task (Arr-Inc), (3) the congruent trials in the color flanker task (Col-Con), and (4) the incongruent trials in the arrow flanker task (Col-Con).

After practice, a block helped the participants to get familiar with the task; 400 trials were presented in four blocks of 100 trials, yielding 100 trials per condition. During each trial, the target stimulus was presented for 500 ms with an intertrial interval of 1,200 ms. Stimuli were presented in blocks of 50 trials separated by short breaks. Each block consisted of an equal amount of the different trial types, with the restriction of a maximum of three times the same stimulus or the same response in succession.

### Electroencephalogram Recordings

The EEG was continuously recorded at a sampling rate of 1,000 Hz with a 19-channel EEG amplifier (the Symtop Instrument®). The recording bandwidth was 0.5 to 100 Hz. The international 10–20 system (FP1, FP2, F3, F4, C3, C4, P3, P4, O1, O2, F7, F8, T3, T4, T5, T6, Fz, Cz, and Pz) was used with linked earlobes as the reference. The electrode impedances were kept below 10 kΩ.

### Data Analyses

#### Behavioral Analyses

The mean reaction times (RTs) of correct response and accuracy were calculated. The results of the RT and the accuracy were submitted to a two-way repeated measures ANOVA using the SPSS 22.0 software. The two within-subject factors were conflict (congruent vs. incongruent) and stimulus type (arrow flanker vs. color flanker). Effect sizes were showed using partial eta square ($\eta_{\text{p}}^{2}$) and Cohen's *d*. Two-tailed paired *t*-tests were applied for pairwise comparisons of behavioral data.

#### ERP Analyses

Based on a previous related literature (Hamm et al., 2002; Di Russo et al., 2003; Mudrik et al., 2010; Fan et al., 2015; Kałamała et al., 2018; Zhou et al., 2019), the following ERP components were selected: (a) anoccipital N1 component (180–220 ms) and a fronto-central P2 component (200–240 ms), (b) a fronto-parieto-central N2b component (260–320 ms), (c) a temporo-parieto-central N300 component (300–360 ms), (d) a parieto-central P3b component (380–440 ms), and (d) a fronto-central N400 component (460–520 ms).

Statistical software (Mindwave-sorting and SPM) was used for the ERP spatiotemporal analysis developed in our lab (application in literature, Zhou et al., 2004, Cao et al., 2017; Cheng et al., 2018; Zhou et al., 2019). EEG data would be pre-processed by MindWave-sorting offline. At first, ocular, muscular, and any other artifacts within the EEG signal were detected at the threshold of ± 70 μV by the MindWave-Sorting, and the EEG signal was automatically corrected *via* a principal component analysis method (Lins et al., 1993a,b). After that, epochs were segmented, ranging from 100 ms before the target stimulus onset and 600 ms after the target stimulus onset. Then, the baseline correction was conducted to correct the pre-stimulus activities. The baseline ERP measurement was the mean amplitude of a 100-ms pre-stimulus interval. At last, we got the grand average waveforms of four trial types by using SPM to perform the total average. Only correct response trials were averaged.

Event-related potentials data at each time point for all channels (electrode-wise) were submitted to two-way repeated measures ANOVA. The correction for multiple testing is based on the false discovery rate procedure (FDR, Benjamini and Yekutieli, 2001; Lage-Castellanos et al., 2010). The two within-subject factors were conflict (congruent vs. incongruent) and stimulus type (arrow flanker vs. color flanker). Two-tailed paired *t*-tests were applied for pairwise comparisons. A multichannel time series of *F*-values/*t*-values were used to generate topographical maps *via* an interpolation method relevant to a generalized cortical imaging technique (Zhou et al., 1998). The statistical parametric mapping (SPM) of *F*-values will be referred to as SPM(F) hereafter. The topographical maps series were derived from the averaged *F*-values within fixed 20 ms windows and a sliding step of 20 ms without overlapping data. The significance threshold was set to 0.05 for all analyses.

## Results

### Behavioral Data

Table 1 presented descriptive statistics of the RT and the accuracy. Table 2 showed the results of the two-way repeated measures ANOVA. The interaction and the main effect of conflict factors and stimulus type factors of the RT and accuracy were all significant. Follow-up pairwise comparisons revealed significant conflict effect (incongruent-congruent) for RT and accuracy in the arrow flanker task [*t*_(24)_ = 9.881, *p* < 0.01, *d* = 1.976], but not in the color flanker task (Table 3). In the congruent trials, RT was significantly lower, and accuracy was obviously higher in the arrow flanker task than the color flanker task [*t*_(24)_ = 8.454, *p* < 0.01, *d* = 1.691; *t*_(24)_ = 7.597, *p* < 0.01, *d* = 1.519]; however, there was no significance between the two types of the task in the incongruent trials.

**Table 1:** Behavioral performance summary (mean ± SD) (*N* = 25).

	**Reaction time (ms)**		**Accuracy (%)**	
	**Arrow**	**Color**	**Arrow**	**Color**
Congruent	440.63 ± 57.23	495.91 ± 72.65	91.98 ± 10.35	84.50 ± 11.24
Incongruent	484.94 ± 68.22	497.61 ± 74.56	85.32 ± 14.75	84.94 ± 9.63

*Arrow, arrow flanker task; color, color flanker task*.

**Table 2:** Two-Factor ANOVA of repeated measures of behavioral data (*N* = 25).

	**Reaction time**			**Accuracy**		
	***F***_**(1, 24)**_	***P***	**$\eta_{\text{p}}^{2}$**	***F***_**(1, 24)**_	***P***	**$\eta_{\text{p}}^{2}$**
Conflict (Inc., Con.)	68.235	0.000	0.740	24.409	0.000	0.504
Task effect (Arr., Col.)	32.753	0.000	0.577	13.019	0.001	0.352
Interaction	85.535	0.000	0.781	18.084	0.000	0.430

*Inc, incongruent; con, congruent; arr, arrow flanker task; col, color flanker task*.

**Table 3:** Conflict effect (Inc.-Con.) of the arrow and color flanker tasks.

**Conflict effect**	**SRC (arrow)**	**SSC (color)**	***t***	***P***	***d***
Reaction time (ms)	44.31 ± 22.42	1.71 ± 12.27	9.249	0.000	1.850
Accuracy (%)	−6.66 ± 6.55	0.44 ± 3.42	−4.253	0.000	−0.851

*Inc, incongruent; con, congruent; SRC, stimulus-response conflict; SSC, stimulus-stimulus conflict*.

### The ERP Waveform and Component Analysis

**Figure 2:**
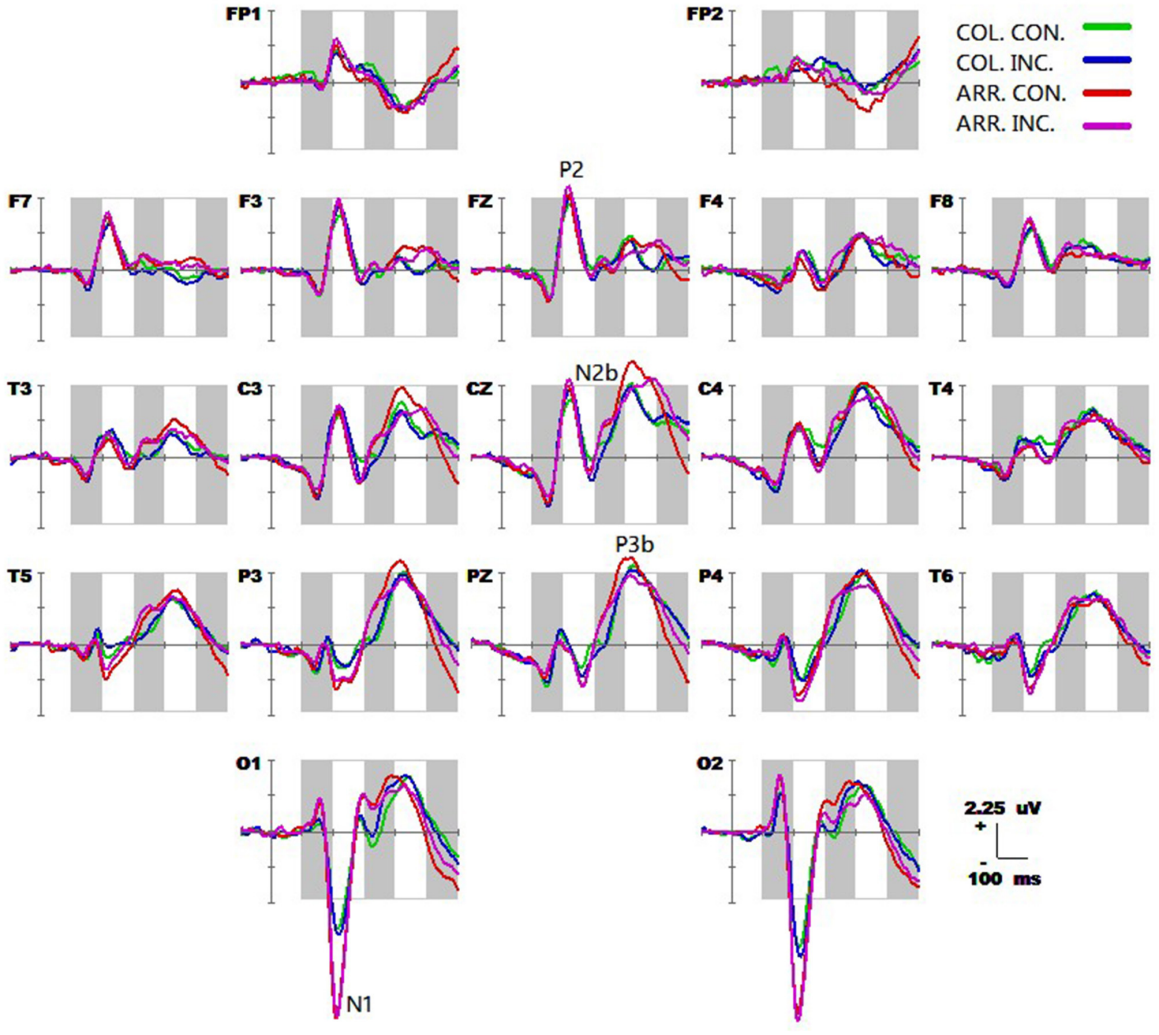
Grand average event-related potential (ERP) waveforms (from −100 to 600 ms) are shown for 19 electrodes across all trial types, from the 25 subjects. The green, blue, red, and purple traces correspond to group average ERP of the color-congruent, color-incongruent, arrow-congruent, and arrow-incongruent conditions, respectively. The baseline ERP measurement is the mean amplitude of a 100-ms pre-stimulus interval.

The grand-average ERP waveforms (from −100 to 600 ms) of all 19 electrodes (FP1, FP2, F3, F4, C3, C4, P3, P4, O1, O2, F7, F8, T3, T4, T5, T6, Fz, Cz, and Pz) are shown in Figure 2. Significant effects were demonstrated for the occipital N1 (200–240 ms), the fronto-central P2 (200–240 ms), the fronto-central N2b (260–320 ms), the temporo-parieto-central N300 (300–360 ms), the parieto-central P3b (380–440 ms), and the fronto-central N400 (460–520 ms) components. The significant average statistics (i.e., *F*-value, $\eta_{\text{p}}^{2}$) of N1, P2, N2b, N300, P3b, and N400 at typical prominent electrodes (O1, O2, Fz, Cz, C3, C4, Pz) within a 20-time window without overlapping data are shown in Tables 4, 5. The measures of N1, P2, N2b, N300, P3b, and N400 with all electrodes are shown in the Supplementary Tables.

**Table 4:** Significant N1, P2, N2b effects (site) within a 20-ms time window (*N* = 25).

**Effect**	***F***_**(1, 25)**_/***p***	**N1(O1)**		**N1(O2)**		**P2(Fz)**		**P2(Cz)**		**N2b(Fz)**		**N2b(Cz)**		**N2b(C3)**		**N2b(C4)**	
	***t***_**(1, 25)**_/***p***, **ES/WO**	**Stat**.	**p/WO**	**Stat**.	**p/WO**	**Stat**.	**p/WO**	**Stat**.	**p/WO**	**Stat**.	**p/WO**	**Stat**.	**p/WO**	**Stat**.	**p/WO**	**Stat**.	**p/WO**
Conflicts	*F/p*	3.29	0.082	4.42	0.051	6.77	0.02	6.50	0.021	5.11	0.042	13.78	0.001	6.38	0.026	17.43	0.000
	$\eta_{\text{p}}^{2}$/WO	0.12	180	0.15	180	0.21	200	0.21	200	0.17	260	0.36	260	0.20	280	0.41	240
Task type	*F/p*	27.46	0.000	20.76	0.000	5.71	0.036	5.51	0.040	6.58	0.040	4.91	0.043	3.56	0.071	2.12	0.168
	$\eta_{\text{p}}^{2}$/WO	0.52	180	0.45	180	0.19	200	0.18	200	0.21	240	0.16	240	0.12	240	0.08	240
Interaction	*F/p*	3.92	0.059	4.24	0.053	0.20	0.659	0.75	0.395	11.69	0.002	0.61	0.008	11.75	0.003	5.63	0.045
	$\eta_{\text{p}}^{2}$/WO	0.14	180	0.15	180	0.01	200	0.03	200	0.32	240	0.26	280	0.32	280	0.18	240

*ES, effect size; WO, window onset*.

**Table 5:** Significant N300, P3b, and N400 effects (site) within a 20-ms time window (*N* = 25).

**Effect**	***F***_**(1, 25)**_/***p***	**N300(Pz)**		**P3b(Cz)**		**P3b(Pz)**		**N400(Fz)**		**N400(Cz)**	
	***t***_**(1, 25)**_/***p***, **ES/WO**	**Stat**.	**p/WO**	**Stat**.	**p/WO**	**Stat**.	**p/WO**	**Stat**.	**p/WO**	**Stat**.	**p/WO**
Conflicts	*F/p*	2.45	0.130	24.06	0.000	10.03	0.005	1.95	0.175	3.36	0.079
	$\eta_{\text{p}}^{2}$/WO	0.09	300	0.49	380	0.29	380	0.07	440	0.12	420
Task type	*F/p*	22.47	0.000	3.65	0.068	1.40	0.248	10.05	0.006	33.23	0.000
	$\eta_{\text{p}}^{2}$/WO	0.47	300	0.13	380	0.05	380	0.29	440	0.57	420
Interaction	*F/p*	1.91	0.180	8.21	0.011	12.09	0.003	0.39	0.538	1.16	0.292
	$\eta_{\text{p}}^{2}$/WO	0.07	300	0.25	380	0.33	360	0.02	440	0.04	420

*ES, effect size; WO, window onset*.

### Spatiotemporal Pattern of ERP: SPM (F) and SPM (t)

**Figure 3:**
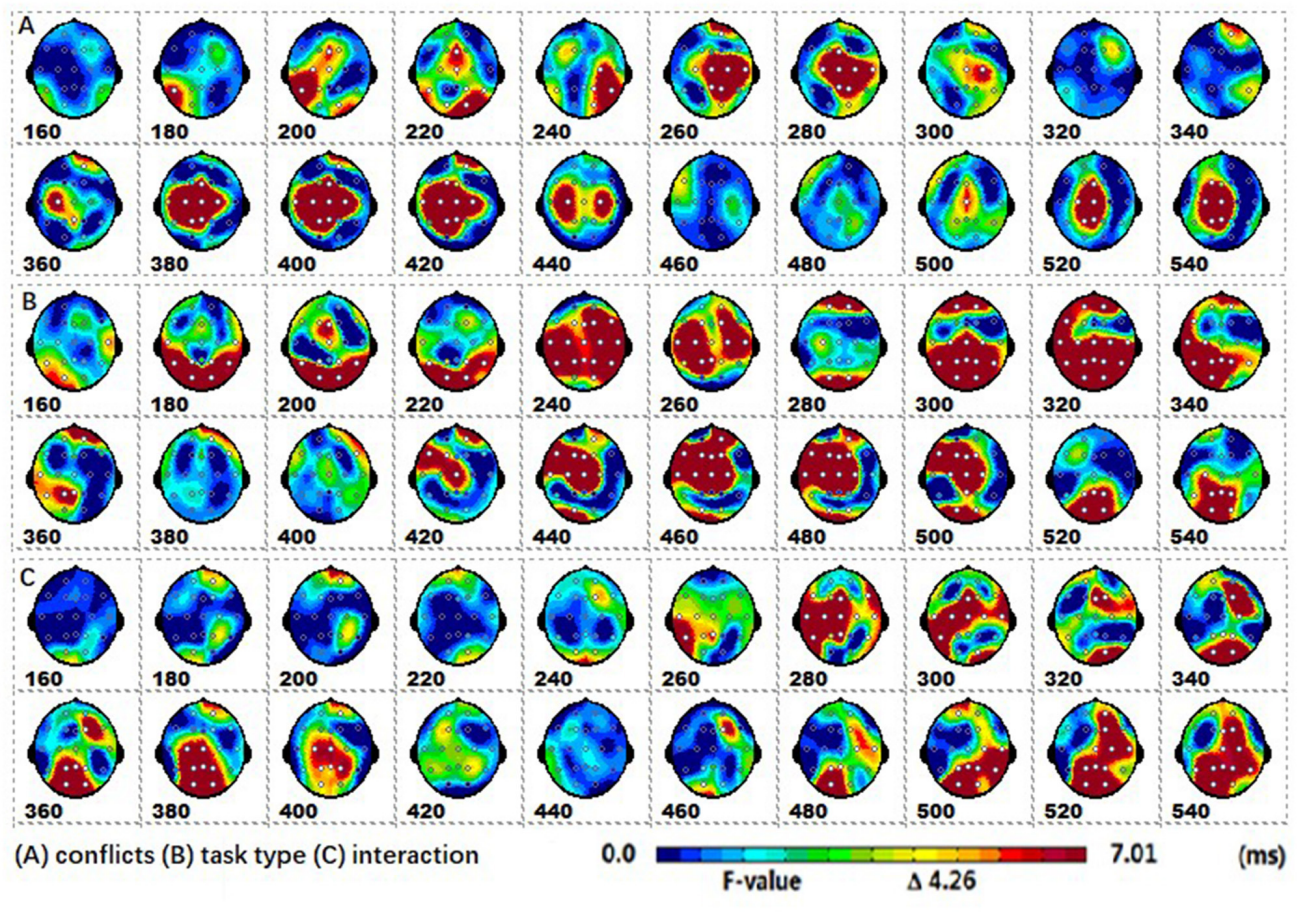
The spatiotemporal patterns of SPM (*F*) (160 to 540 ms) are derived from the two-way (conflict: congruent vs. incongruent, and stimulus type: arrow flanker vs. color flanker) repeated measures ANOVA: **(A)** the conflict effect, **(B)** the task type effect, and **(C)** the interaction effect. Each map was interpolated from the average *F*-values within the fixed 20-ms time window, and the bright yellow bin of the color scale corresponded to the.05 significance threshold: *F*_(1, 24)_ = 4.26. The white dots represented the electrode sites with significant effects.

The spatiotemporal patterns of SPM (F) (160–540 ms) derived from the two-way repeated measures ANOVA are shown in Figure 3. Each map was interpolated from the average *F*-values within the fixed 20 ms time window, and the bright yellow bin of the color scale corresponded to the 0.05 significance threshold: *F*_(1, 24)_ = 4.26. The white dots represented the electrode sites with significant effects. As described in Figure 2, (A) the conflict factor induced the fronto-central P2, the fronto-parieto-central N2b, and the parieto-central P3b; (B) the task type factor involved the occipital N1, the frontal-central P2, the fronto-central N2b, the fronto-parieto-central N300, and the fronto-central N400; (C) The interaction effect was present at the fronto-central and left fronto-parieto-central (N2b), and the parieto-occipital (P3b) regions.

**Figure 4:**
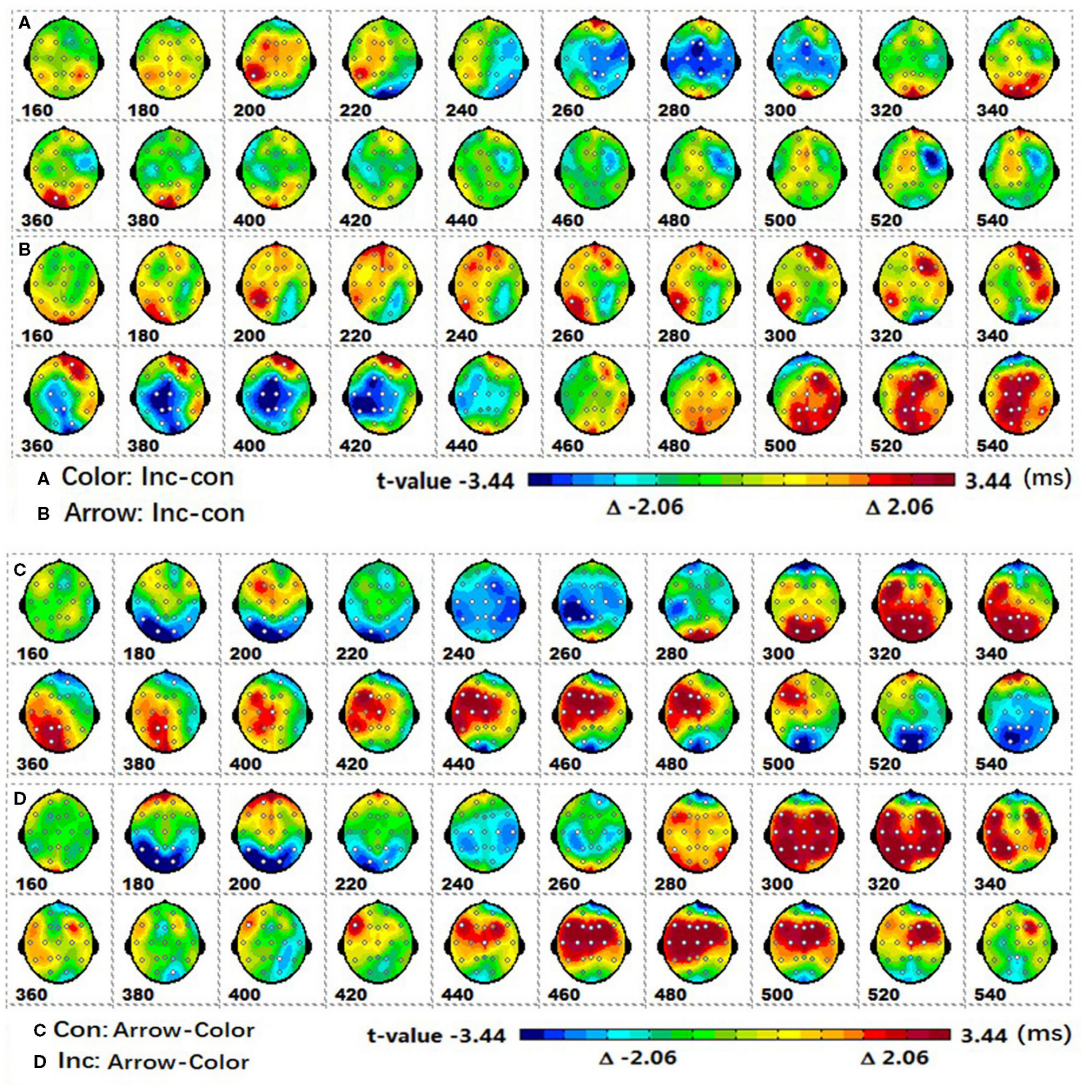
The spatiotemporal patterns of SPM (*t*) from 160 to 540 ms are derived from the pairwise comparisons between the conditions (Col, Arr, Con, Inc): **(A)** (Col.Inc-Col.Con), **(B)** (Arr.Inc-Arr.Con), **(C)** (Con.Arr-Con.Col), **(D)** (Inc.Arr-Inc.Col). Each map is interpolated from the average *t*-values within window length of 20 ms; the white dots represent the sites with significant effects. For the spatiotemporal patterns of SPM (*t*), the colors beyond the 0.05 significant threshold *t*_(24)_ = 2.06 at the two ends of the color scale represent significant regions. Col, color; Arr, arrow; Con, congruent; Inc, incongruent.

*Post-hoc* tests revealed that (1) the incongruent condition elicited a larger fronto-central P2 and fronto-parieto-central N2b than congruent condition, whereas the parieto-central P3b was significantly more positive for congruent than incongruent conditions; (2) the arrow flanker task evoked a more negative occipital N1, and the color flanker induced a fronto-parieto-central N2b/N300 and fronto-central N400, see Figures 4A–D.

## Discussion

The purpose of this study was to separate the neural correlates of SSC and SRC and parse the neural mechanism of conflict effects based on STM or LTM in flanker tasks. In the present study, the color flanker task may mainly contain the color category effect and SSC based on STM, while the arrow flanker task mainly contained SRC based on LTM.

The interaction effects and main effects of conflicts and task types in this study are significant. Among them, the arrow task has a faster reaction time and a higher accuracy rate than the color task, which is consistent with the literature (Peschke et al., 2013). The possible reason is that the arrow is an overlearning stimulus symbol, which can be processed automatically. The behavioral performance of conflicting trials is worse than that of congruent trials. Furthermore, pair-wise comparison analysis showed that the reaction time difference of the arrow task is significant (SRC effect), which is consistent with the previous flanker studies (Brown and Besner, 2001; Noyce and Sekuler, 2014). However, the reaction time difference of the color task is not significant (SSC effect), which is inconsistent with the previous flanker study (Van Veen and Carter, 2002; Zhou et al., 2019). It is incredible that no SSC effect was observed in the color flanker task. Many previous studies on perceptual flanker task have demonstrated the existence of the SSC effect (Van Veen and Carter, 2002; De Houwer, 2003; Frühholz et al., 2011; Mansfield et al., 2013), even in our own study using a color flanker 2–1 mapping task (Zhou et al., 2019). To investigate the reason for the lack of SSC effect in the color flanker task, we conducted a *post-hoc* analysis on ERP data of the color flanker task between incongruent condition and congruent condition. The results showed that incongruent trials in the color flanker task induce a larger frontal-central N2b than the congruent trials, and reflected the process of conflict resolution (Sohn et al., 2007; Kim et al., 2011; see details below), which suggests the existence of SSC effect in the color flanker task. The SSC effect in the color flanker task was observed in ERP data but lacks in behavioral performance. After a detailed comparison with the experimental design of the previous literature using the color flanker task (De Houwer, 2003; Zhou et al., 2019), we supposed that the possible reason for the absence of SSC effect in behavioral performance was that the more amount of conflict effect was induced by multiple types of stimulus attributes in previous studies than the current study with just two colors.

Previous studies on which ERP components are involved in SSC and SRC are still controversial, and our results provided some evidence for this issue. The SSC involved in the color task caused the N2b enhancement effect in the frontal central area (260–320 ms) in this study. This is consistent with previous literature (Forster et al., 2011; Zhou et al., 2019). The N2b component has always been considered to be involved in conflict resolution and might correspond to the activation of ACC during the conflict evaluation stage (Van Veen and Carter, 2002; Sohn et al., 2007; Kim et al., 2011). The SRC contained in the arrow task leads to two ERP effects. First of all, the incongruent trials have enhanced the central area P2 (200–240 ms), compared with the congruent trials. This result is consistent with the application of arrow flanker tasks (Kałamała et al., 2018). Research using the combined task of flanker and Simon also reported a similar P2 effect (Korsch et al., 2016). Frontal area P2 generally reflects the selective attention process (Luck and Hillyard, 1994). The enhanced effect of frontal central P2 suggests that the resolution of SRC involves the early selective attention process. Second, the incongruent trials weakened the top center P3b (380–440 ms) compared with the congruent trials. The decrease of P3b in the top central area might reflect resource competition and restraint control (Kok, 2001; Polich, 2007). Incongruent trials need to restrain competing reaction codes more than consistent trials, resulting in lower P3b.

The color and the arrow flanker task contain the SSC based on STM and SRC based on LTM, respectively. The results of this study showed the increased fronto-central N2b effect was observed in the color flanker task and reflected the resolution of SSC based on STM, while the SRC based on LTM in the arrow flanker task was involved in the fronto-central P2 effect and the parieto-central P3b effect. The fronto-central N2b effect induced by SSC was in line with previous studies (Hillman et al., 2009; Frühholz et al., 2011; Zhou et al., 2019). A recently published paper that employed a 2:1 mapping color flanker task reported that SSC caused the enhanced fronto-central N2b effect (Zhou et al., 2019). Similar results were reported in the perceptual flanker task (Forster et al., 2011). Forster et al. performed an ERP study using the letter flanker task and revealed increases in the frontal-central N2b amplitudes in incongruent trials, and decreases in the N2b amplitudes with increasing incongruity. The above results suggested that the increased fronto-central N2b effect was involved in conflict processing of SSC based on STM and may correspond to the activation of the anterior cingulate cortex during conflict evaluation (Van Veen and Carter, 2002; Sohn et al., 2007; Kim et al., 2011). However, some studies using the arrow flanker task, also reported the similar N2b effect (Acheson and Hagoort, 2014; Bailey et al., 2016; Korsch et al., 2016; Olson et al., 2016; Pan et al., 2020), which makes it possible for the N2b effect to involve in the resolution of SRC based on LTM. For example, the frontal-central N2b effect was reported by Korsch et al., who found that the N2b amplitude is increased in incongruent compared with congruent trials in a combined Flanker conflict and a stimulus-response-conflict (SRC) task (Korsch et al., 2016). An ERP study using the arrow flanker task conducted by Pan et al. (2020) also showed the enhanced N2 effect in incongruent trials, compared with congruent trials. Nevertheless, The N2s reported in previous studies using arrow flanker tasks, vary substantially in their topographical and temporal characteristics and do not seem to correspond to the conflict N2 component, which reflected the process of conflict resolution. Moreover, a large sample of participants study that employed the arrow flanker task to specifically investigate the presence of the conflict N2 component in the flanker paradigm also reported that no enhanced N2 (also called “N2b”) effect was observed in incongruent trials compared with congruent trials (Kałamała et al., 2018), and the author proposed that the absence of the conflict N2 in the arrow flanker task indicated that response inhibition may not be crucial to the resolution of conflict induced by incongruent flankers (SRC based on LTM). This is consistent with our study: the arrow flanker stimuli did not cause the enhanced fronto-central N2b effect. In consequence, based on the above evidence, we maintained that the conflict processing of SRC based on LTM did not involve the N2b component.

The results of the present study also demonstrated that the fronto-central P2 and the parieto-central P3b effect were involved in the resolution of SRC based on LTM in the arrow flanker task. As discussed above, the increased frontal-central P2 effect has also been reported in studies using the arrow flanker task (Korsch et al., 2016; Kałamała et al., 2018), and might reflect the selective attention process (Luck and Hillyard, 1994). The latency (i.e., 200–240 ms) and scalp distribution (i.e., dorsolateral frontal region) of the P2 effect in our study were consistent with the study by Kałamała et al. (2018) and Korsch et al. (2016) suggesting that the enhanced frontal-central P2 might indicate the process of selective attention and seem to involve in the resolution of SRC based on LTM. Another ERP component involved in the SRC effect was the parieto-central P3b, that is, the incongruent trials in the arrow flanker task elicited smaller P3b than the congruent trials. This is in line with the application of arrow flanker tasks. An ERP study employing a combined Flanker conflict and the SRC task also revealed that the Flanker conflict conditions were associated with significantly reduced P3 amplitude (Korsch et al., 2016). The P3b component with parieto-central topography has been suggested to be an association with the allocation of attentional resources and the process of inhibition control, particularly related to the suppression of irrelevant stimuli (Kok, 2001; Neuhaus et al., 2010). The decreased P3 amplitude in incongruent trials might indicate that individuals consume more attentional resources due to higher task demands. As a consequence, the P3b effect observed in the arrow flanker task appeared that resource competition and restraint control might play an important role in resolving the conflict of SRC based on LTM. However, it is noted that even the same type of conflict based on different types of memory associations may involve different neural mechanisms. The most obvious evidence was the study conducted by Zhou et al. (2019). They employed a 2-1 mapping flanker task to separate stimulus conflict and response conflict and demonstrated that the SRC based on STM induced the N2b effect rather than the P3b effect. Besides, both SSC and SRC based on STM enhance the frontal P2, and the increased effect was larger for SRC.

In addition, when performing the color and arrow flanker task, in addition to solving SSC and SRC, subjects may also involve the process of a color category or symbol processing. Firstly, compared with the color stimulation, the arrow stimulation caused the N1 enhancement effect in the occipital area (180–240 ms). Previous studies have shown that the brain region with a specific response to visual vocabulary involved the activation of visual word form area (VWFA) (Dehaene and Cohen, 2011; Lerma-Usabiaga et al., 2018; Chen et al., 2019), and evoked a negative wave (N170) in the occipito-temporal region about 100–250 ms post-stimulus (Fan et al., 2015). Since the arrows in the flanker task were the visual graphic stimulus, the enhancement effect of occipital area N1 might suggest that the arrow symbols have been specially processed in the early stage and SRC is processed by the symbol system. The reaction time of arrow symbols is generally significantly shorter than that of color stimuli, which also supports faster symbol processing. Secondly, compared with the arrow stimulus, the color stimulus successively elicited the frontal top N2b/N300 component (300–360 ms) and the frontal central N400 component (460–520 ms). The N300 is acknowledged to reflect pre-semantic perceptual processes (Schendan and Maher, 2009; Mudrik et al., 2010). In a study exploring the N300 and N400 effects with picture stimuli in congruent or incongruent contents (Hamm et al., 2002), the N300 effect only appeared in the case of between-category mismatches, suggesting that it reflected the strategy classification process. The color flanker incongruent trials were also a type of between-category mismatches; the fronto-parieto-central N300 observed in our study might reveal the classification of picture perception. Several studies have demonstrated that the N400 was related to semantic memory processing, and it was not only induced by words but also stimuli, such as pictures and sounds (Kutas and Federmeier, 2000, 2011). Ganis et al. applied the words and pictures with a similar meaning to investigate the brain processes, subserving the different types of stimuli, and found that pictures elicited a similar but more frontally distributed N400, similar to that for concrete words (Ganis et al., 1996). This N400 effect was also observed in other studies using non-word stimuli (Van Petten and Rheinfelder, 1995; Olivares et al., 1999). In the present study, different color picture stimuli correspond to different reactions; subjects need to classify the colors and activate the correct stimulus-response mapping before a response. Therefore, the N400 effect induced by the task type factors might suggest the picture naming effect. Overall, the N300 and N400 cue color stimulation observed in this experiment activates the color picture processing system. After the SSC is resolved, the color stimuli still undergo the process of image classification activation.

In this study, the following limitations should be addressed in future studies. First, although the influence of graphic information carried by the stimulus itself was excluded when comparing different conflict processes in this experiment, the SSC and SRC effects in the arrow Flanker task were not strictly separated. Therefore, a better design is needed to compare the differences between SSC and SRC in a flanker task in the future, such as employing oriented hands and arrows in a flanker paradigm. Second, the sparse electrode sampling used in the present study is difficult to provide precise source estimation. Further source localization studies with high-density EEG sensors will improve our understanding of neural cognitive networks underlying cognitive control.

The present study revealed that the SSC based on STM induced in the color flanker task enhances fronto-central N2b, and reflected the process of conflict evaluation and resolution. The SRC based on LTM caused in the arrow flanker task successively enhanced frontocentral P2 but reduced centroparietal P3b, suggesting that solving SRC based on LTM involves early selective attention and later inhibition of motor commands that are automatically activated. Moreover, the arrow flanker task enhances the occipital N1, suggesting that it is specially processed by the symbol system, and the color stimuli successively elicit the parietal N300 and the fronto-central N400, indicating that it is involved in the process of color picture classification activation. In conclusion, these findings implied that conflict effects based on different types of memory associations involve different neural mechanisms; SSC based on STM involves in N2b effect, while SRC based on LTM involves in P2 and P3b effect.

## Data Availability Statement

The original contributions presented in the study are included in the article/Supplementary Material, further inquiries can be directed to the corresponding author/s.

## Ethics Statement

The studies involving human participants were reviewed and approved by the Medical Ethics Committee of Nanfang Hospital, Southern Medical University. The patients/participants provided their written informed consent to participate in this study.

## Author Contributions

WC, QH, YC, SZ, and ZC contributed to the conception and design of the study and writing of the manuscript. WC, WD, LC, and SS acquired the recording data. WC, QH, and YC performed the analysis and prepared all the figures and tables. All the authors reviewed the main manuscript text.

## Conflict of Interest

The authors declare that the research was conducted in the absence of any commercial or financial relationships that could be construed as a potential conflict of interest.

## Publisher's Note

All claims expressed in this article are solely those of the authors and do not necessarily represent those of their affiliated organizations, or those of the publisher, the editors and the reviewers. Any product that may be evaluated in this article, or claim that may be made by its manufacturer, is not guaranteed or endorsed by the publisher.
