# Supplementary material for: Parsing the Neural Mechanisms of Short-Term and Long-Term Associations in the Flanker Tasks: An ERP Analysis
Source: Front Behav Neurosci. 2021 Aug 5;15:626907. doi: 10.3389/fnbeh.2021.626907 (PMC8374427; doi:10.3389/fnbeh.2021.626907)
Supplement: Supplementary file 1 [file Data_Sheet_1.PDF]

Table 1 Summary of N1 amplitude for four trial types

Electrode	N1 (μV)			
	Con-Arr	Inc-Arr	Con-Col	Inc-Col
FP1	-	-	-	-
FP2	-	-	-	-
F7	-0.92	-0.79	-0.92	-1.21
F8	-0.92	-0.86	-0.93	-1.12
F3	-1.41	-1.50	-1.52	-1.41
F4	-1.14	-0.90	-1.02	-1.42
C3	-2.43	-2.02	-2.42	-2.51
C4	-1.59	-1.72	-1.93	-2.15
P3	-1.45	-1.05	-1.80	-1.53
P4	-0.75	-0.71	-1.27	-0.99
O1	-11.39	-11.22	-6.01	-6.39
O2	-11.05	-11.55	-7.12	-7.73
T3	-1.41	-1.06	-1.11	-1.45
T4	-1.31	-1.03	-1.09	-1.44
T5	-0.94	-0.56	-0.93	-0.78
T6	-0.59	-0.24	-0.85	-0.97
Fz	-1.86	-1.68	-1.60	-1.78
Cz	-2.80	-2.38	-2.95	-2.97
Pz	-2.06	-1.77	-2.58	-2.35

Con: congruent; Inc: incongruent; Arr: arrow flanker task; Col: color flanker task; -: no corresponding ERP component on this electrode.

Table 2 Summary of P2 amplitude for four trial types

Electrode	P2 (μV)			
	Con-Arr	Inc-Arr	Con-Col	Inc-Col
FP1	-	-	-	-
FP2	-	-	-	-
F7	3.24	3.57	2.94	2.87
F8	3.06	3.21	2.52	2.68
F3	4.14	4.31	3.42	3.96
F4	0.66	1.18	1.12	1.19
C3	2.81	3.19	2.66	3.16
C4	2.16	1.88	1.80	2.00
P3	-0.07	0.02	0.27	0.40
P4	0.57	0.48	0.13	0.48
O1	-	-	-	-
O2	-	-	-	-

T3	1.17	1.53	1.51	1.73
T4	0.73	0.81	1.14	1.18
T5	0.10	0.36	0.52	0.92
T6	0.22	0.62	0.45	0.63
Fz	4.64	6.17	3.75	4.52
Cz	4.42	4.80	3.57	4.24
Pz	0.12	0.25	0.68	0.93

Con: congruent; Inc: incongruent; Arr: arrow flanker task; Col: color flanker task; -: no corresponding ERP component on this electrode.

Table 3 Summary of N2b amplitude for four trial types

Electrode	N2b (μV)			
	Con-Arr	Inc-Arr	Con-Col	Inc-Col
FP1	-	-	-	-
FP2	-	-	-	-
F7	-0.02	0.33	0.20	-0.28
F8	-0.16	-0.01	0.37	-0.12
F3	-0.69	-0.57	-0.15	-0.88
F4	-1.28	-0.87	-0.66	-1.07
C3	-1.56	-1.51	-0.33	-1.38
C4	-0.49	-0.88	0.76	-0.55
P3	-	-	-	-
P4	-	-	-	-
O1	-	-	-	-
O2	-	-	-	-
T3	-0.75	-0.40	0.04	-0.69
T4	-0.31	-0.35	0.47	-0.03
T5	-	-	-	-
T6	-	-	-	-
Fz	-0.38	-0.40	0.28	-0.61
Cz	-0.81	-1.02	-0.10	-1.38
Pz	-	-	-	-

Con: congruent; Inc: incongruent; Arr: arrow flanker task; Col: color flanker task; -: no corresponding ERP component on this electrode.

Table 4 Summary of N300 amplitude for four trial types

Electrode	N300 (μV)			
	Con-Arr	Inc-Arr	Con-Col	Inc-Col
FP1	-	-	-	-
FP2	-	-	-	-
F7	0.14	0.70	0.16	-0.25
F8	0.63	1.01	0.81	0.52
F3	0.10	0.04	-0.41	-0.13
F4	-0.10	0.15	-0.26	-0.36
C3	1.12	1.45	0.22	0.13
C4	2.48	2.83	2.14	1.52
P3	1.85	1.94	0.31	0.39
P4	-	-	-	-
O1	-	-	-	-
O2	-	-	-	-
T3	1.03	1.02	0.02	0.08
T4	1.81	2.27	1.73	1.65
T5	-	-	-	-
T6	-	-	-	-
Fz	-0.11	0.12	0.56	0.44
Cz	2.20	2.41	0.98	1.24
Pz	1.86	1.71	0.02	-0.11

Con: congruent; Inc: incongruent; Arr: arrow flanker task; Col: color flanker task; -: no corresponding ERP component on this electrode.

Table 5 Summary of P3b amplitude for four trial types

Electrode	P3b (μV)			
	Con-Arr	Inc-Arr	Con-Col	Inc-Col
FP1	-	-	-	-
FP2	-	-	-	-
F7	-	-	-	-
F8	-	-	-	-
F3	1.53	1.31	0.87	0.75

F4	2.21	2.26	2.29	2.28
C3	4.40	2.87	3.47	2.96
C4	4.67	3.87	4.64	4.37
P3	5.26	4.17	4.58	4.42
P4	4.58	3.73	4.47	4.70
O1	3.55	2.98	3.47	3.56
O2	3.19	2.38	2.87	3.17
T3	2.40	1.73	1.71	1.46
T4	2.71	2.36	3.09	2.98
T5	3.45	3.01	2.88	2.95
T6	2.79	3.01	3.32	3.23
Fz	1.93	1.88	2.13	1.80
Cz	6.01	4.98	4.59	4.43
Pz	5.46	4.37	4.98	4.70

Con: congruent; Inc: incongruent; Arr: arrow flanker task; Col: color flanker task; -: no corresponding ERP component on this electrode.

Table 6 Summary of N400 amplitude for four trial types

Electrode	N400 (μV)			
	Con-Arr	Inc-Arr	Con-Col	Inc-Col
FP1	-1.93	-1.58	-1.58	-1.68
FP2	-1.80	-0.75	-0.70	-0.35
F7	-	-	-	-
F8	-	-	-	-
F3	1.33	0.59	-0.23	-0.27
F4	0.93	1.59	0.68	0.35
C3	3.38	2.81	1.40	1.29
C4	-	-	-	-
P3	-	-	-	-
P4	-	-	-	-
O1	-	-	-	-
O2	-	-	-	-
T3	-	-	-	-
T4	-	-	-	-
T5	-	-	-	-
T6	-	-	-	-
Fz	1.56	1.54	0.02	-0.03
Cz	3.18	4.26	2.17	2.39
Pz	-	-	-	-

Con: congruent; Inc: incongruent; Arr: arrow flanker task; Col: color flanker task; -: no corresponding ERP component on this electrode.
